# Unveiling the Bioactive Potential of the Invasive Jellyfish *Phyllorhiza punctata* Through Integrative Transcriptomic and Proteomic Analyses

**DOI:** 10.3390/biom15081121

**Published:** 2025-08-04

**Authors:** Tomás Rodrigues, Ricardo Alexandre Barroso, Alexandre Campos, Daniela Almeida, Francisco A. Guardiola, Maria V. Turkina, Agostinho Antunes

**Affiliations:** 1 CIIMAR/CIMAR—Interdisciplinary Centre of Marine and Environmental Research, University of Porto, Terminal de Cruzeiros do Porto de Leixões, Av. General Norton de Matos, s/n, 4450-208 Porto, Portugal; tomasfcr.porto@gmail.com (T.R.); barrosoalex98@gmail.com (R.A.B.); amoclclix@gmail.com (A.C.); 2 Department of Biology, Faculty of Sciences, University of Porto, Rua do Campo Alegre 687, 4169-007 Porto, Portugal; 3 Department of Zoology and Physical Anthropology, Faculty of Biology, University of Murcia, Campus of International Excellence, Campus Mare Nostrum, 30100 Murcia, Spain; daniela.martins@um.es; 4 Immunobiology for Aquaculture Group, Department of Cell Biology and Histology, Faculty of Biology, Regional Campus of International Excellence “Campus Mare Nostrum”, University of Murcia, 30100 Murcia, Spain; faguardiola@um.es; 5 Department of Biomedical and Clinical Sciences, Faculty of Medicine and Clinical Sciences, Linköping University, 581 83 Linköping, Sweden; maria.turkina@liu.se

**Keywords:** Phyllorhiza punctata, jellyfish, proteomics, antimicrobial peptides (AMPs), toxins, venom, bioactive compounds, transcriptomics, invasive species, cnidaria, marine biology, deep learning

## Abstract

The white-spotted jellyfish, *Phyllorhiza punctata*, is an invasive species with significant ecological and economic relevance spreading across various regions. While its ecological impact is well-documented, its molecular and biochemical characteristics remain poorly understood. In this study, we integrate proteomic data generated by LC-MS/MS with publicly available transcriptomic information to characterize *P. punctata*, analyzing differential protein expression across three distinct tissues: oral arms, mantle, and gonads. A total of 2764 proteins and 25,045 peptides were identified, including several venom components such as jellyfish toxins (JFTs) and phospholipase A2 (PLA2), which were further investigated and compared to toxins from other species. Enrichment analyses revealed clear tissue-specific functions. Additionally, deep learning and machine learning tools identified 274 promising AMP candidates, including the α-helical, β-sheet, and αβ-motif peptides. This dataset provides new insights into the protein composition of *P. punctata* and highlights strong AMP candidates for further characterization, underscoring the biotechnological potential of underexplored cnidarian species.

## 1. Introduction

Jellyfish (phylum Cnidaria) are an ancient and diverse group of gelatinous zooplankton predominantly found in marine ecosystems, where they play important ecological roles [1,2]. Their ability to sting through the deployment of specialized stinging cells called nematocysts [3] and their association with jellyfish blooms are their most well-known characteristics. These blooms are sudden massive population increases that can disrupt ecosystems by altering nutrient dynamics, outcompeting native species, and interfering with local food chains. Moreover, they negatively impact human industries such as fishing, tourism, and aquaculture [2,4,5]. Despite these issues, jellyfish remain essential components of marine food webs, playing a crucial role in the dynamics of these ecosystems. They serve as predators of planktonic organisms, crustaceans, small fish, and the eggs and larvae of various marine species [1,6]. Conversely, they are prey for fish, sea turtles, sea slugs, and birds [7,8]. Jellyfish also contribute to nutrient cycling, as they assimilate nutrients during feeding and release inorganic nutrients through excretion and decomposition [9,10]. Given their versatility and ecological importance, jellyfish are increasingly recognized as valuable bioresources. They are used as human food [11], as aquafeed for fish and crustaceans [12], as attractions in zoos and aquariums [13], and as sources of bioactive compounds for biotechnological applications in pharmaceuticals, nutraceuticals, and cosmeceuticals [14].

*Phyllorhiza punctata* von Lendenfeld, 1884 (Cnidaria: Rhizostomeae: Mastigiidae), commonly known as the Australian white-spotted jellyfish, has become a subject of concern due to its invasive nature and expanding distribution. Originally from the South Pacific Ocean, this species has spread to various regions, including the Indian Ocean [15], Caribbean Sea [16], Gulf of Mexico [17], Mediterranean Sea [18], and Northeastern Atlantic Ocean [19]. The rise in sightings of this jellyfish correlates with global changes such as ocean acidification, global warming, and salinity fluctuations [20]. Research on *P. punctata* has largely concentrated on its ecological aspects (e.g., life cycle, distribution, feeding behavior, and invasiveness) [21]. However, there is a gap in studies focusing on its molecular and biochemical characteristics. Some research has examined its lipid composition and extract properties [22]. Moreover, the venom composition of the jellyfish remains poorly understood, with only one article analyzing a crude protein extract using High-Performance Liquid Chromatography (HPLC) [23].

Investigating the proteomic profiles of different tissues, such as the gonads, mantle, and oral arms, could reveal valuable insights into the general protein content of the species, as well as the interesting bioactive compounds.

Recent advancements in computational models have significantly impacted biological sequence analysis, particularly in AMP prediction. These models use various machine learning and deep learning algorithms to analyze peptide sequences and predict their antimicrobial properties. Traditional machine learning methods, based on algorithms such as random forests (RFs), support vector machines (SVMs), and artificial neural networks (ANNs), have been widely used to predict AMPs based on their physicochemical properties, amino acid composition, and sequence motifs, as exemplified by resources like CAMPR4 [24]. More recently, deep learning methodologies, including convolutional neural networks (CNNs), recurrent neural networks (RNNs), and transformer-based models, have improved AMP prediction by effectively capturing complex sequence patterns and structural features, as illustrated by platforms such as AI4AMP [25], AMPScanner vr.2 [26], and PepNet [27]. These computational tools accelerate the discovery of AMPs and help identify potential candidates for therapeutic applications in areas like aquaculture, human medicine, and biotechnology [28]. Despite their advantages, these predictive models show variability in their accuracy, sensitivity, and generalization capabilities, highlighting the need for comparative performance studies to evaluate their reliability [29].

Thus, this study aims to combine transcriptomic and proteomic data, together with computational tools, to explore the bioactive potential of *P. punctata*. By utilizing publicly accessible RNA-seq data, proteomic analysis by mass spectrometry, and computational prediction tools, this research identifies interesting bioactive compounds, including putative toxins and candidate peptides with potential antimicrobial properties that could serve different biotechnological applications.

## 2. Materials and Methods

### 2.1. Sample Collection and Tissue Preparation

**Figure 1:**
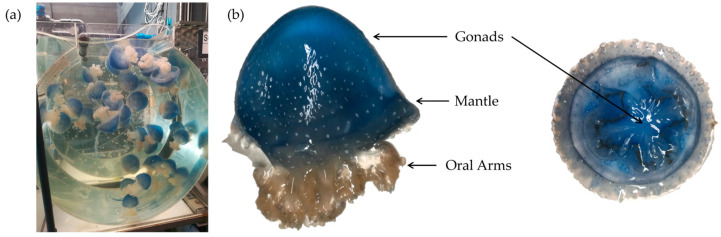
Overview of *P. punctata* specimens and sampled tissues. (**a**) Live specimens maintained in an aerated, circulating aquarium. (**b**) Frontal (**left**) and dorsal (**right**) views of a single specimen, illustrating the sampled tissues and highlighting the characteristic x-shaped gonads.

Three adult specimens of the jellyfish *P. punctata* were provided and collected in March 2022 from the Oceanário de Lisboa (Figure 1). They were maintained in aerated, circulating aquariums at 25 °C, with a salinity of 33.5–34.0, pH of 7.9–8.10, ammonia of 0–0.10 mg/L, nitrites of 0.050–0.150 mg/L, and nitrates of 1.0–10.0 mg/L. These water quality parameters follow established aquaculture guidelines, under routine water exchanges, as outlined in the Jellyfish Care Manual [30]. Oral arms, gonads, and mantle tissues were dissected on-site and immediately placed on dry ice for transport to the laboratory, where they were stored at −80 °C for subsequent analyses.

### 2.2. Protein Extraction and Filter-Aided Sample Preparation

Samples were kept on ice to thaw and then incubated in SDT buffer (2% SDS, 0.1 M Tris/HCl pH 7.6, and 0.1 M dithiothreitol) at a ratio of 0.5 g fresh weight per mL. A protease inhibitor cocktail (Halt PI Cocktail, Cat No. 78429, Thermo Fisher Scientific, Waltham, MA, USA) was added at a 1:100 dilution. The mixture was incubated in the dark at room temperature for 20 min. Following incubation, samples were subjected to sonication for a total of 1 min, with 10-s pulses at an amplitude of 15 µm, using an MSE Soniprep 150 sonicator. Subsequently, the samples were heated to 95 °C for 3 min and then centrifuged at 16,000× *g* for 1 h at room temperature. The supernatants were collected and transferred into new Eppendorf tubes, and the total protein concentration was quantified by absorbance at 280 nm using a NanoDrop One spectrophotometer (Thermo Fisher Scientific, Waltham, MA, USA). The extracted protein samples were stored at −20 °C until further use.

The Filter-Aided Sample Preparation (FASP) method was performed as described by Wiśniewski et al. (2009) [31] with modifications. Protein samples were diluted to 1 μg/μL in SDT buffer. A total of 30 μL of the diluted protein sample was mixed with 200 μL of UA buffer (8 M urea in 0.1 M Tris/HCl, pH 8.5) and transferred to pre-washed filter units (Merck Millipore Amicon Ultra 0.5 mL, Ultracel 10K, Cat. No. UFC501096, Merck Millipore, Darmstadt, Germany). The mixture was centrifuged at 14,000× *g* for 20 min. The flow-through was discarded, and 100 μL of iodoacetamide solution (0.05 M iodoacetamide in UA buffer) was added, mixed at 600 rpm for 1 min using a Thermomixer, and incubated in the dark at room temperature for 20 min. The filters were then centrifuged at 14,000× *g* for 20 min. Three washes were performed with 100 µL UA buffer, then centrifuging conducted at 14,000× *g* for 15 min each. Additional washes were performed using 100 µL of 0.05 M ammonium bicarbonate, then centrifuging conducted at 14,000× *g* for 10 min each. Peptide digestion was carried out by adding trypsin (Roche, recombinant, proteomics grade, Cat No. 3708985001, Mannheim, Germany) at a 1:100 enzyme-to-protein ratio in 0.05 M ammonium bicarbonate. Samples were mixed at 600 rpm for 1 min in a Thermomixer and incubated in a wet chamber at 37 °C for 16 h. Digested peptides were eluted into new collection tubes by centrifugation at 14,000× *g* for 10 min, and a second elution step was performed using 0.5 M NaCl, followed by centrifugation at 14,000× *g* for 10 min. The peptides were acidified with trifluoroacetic acid (TFA, 10% *v*/*v*) to a pH of 2–3. Peptides were desalted using C18 columns (Thermo Fisher Scientific Pierce C18 Tips, 100 µL, Cat. No. 87784, Thermo Fisher Scientific, Waltham, MA, USA), and conditioned with 80% acetonitrile (*v*/*v*) and 0.1% formic acid (*v*/*v*). The acidified samples were loaded onto the columns, washed with 0.1% formic acid (*v*/*v*), and eluted with 80% acetonitrile (*v*/*v*) and 0.1% formic acid (*v*/*v*) into new tubes. Peptide concentration was measured at 280 nm, and samples were dried using a vacuum concentrator (Eppendorf Concentrator Plus, Eppendorf, Hamburg, Germany). Dried peptides were stored at −20 °C until further analysis.

### 2.3. LC-MS Analysis of Protein Samples

LC-MS/MS analysis was performed using a nano-LC coupled to a Q Exactive HF Hybrid Quadrupole-Orbitrap Mass Spectrometer (Thermo Fisher Scientific, Waltham, MA, USA). Peptides were separated by reverse-phase chromatography on an EASY-Spray C18 reverse-phase nano-LC column (PepMap RSLC C18, 2 µm, 100A 75 µm × 25 cm, Thermo Fisher Scientific) with a gradient of 0.1% formic acid in water (A) and 0.1% formic acid in 80% acetonitrile (B) as follows: from 6% B to 30% B in 65 min; from 30% B to 100% B in 20 min; and 100% B from 85 to 90 min, at a flow rate of 0.3 µL/min. Separated peptides were electrosprayed and analyzed on a Q Exactive HF Mass Spectrometer (Thermo Fisher Scientific, Waltham, MA, USA) in a positive polarity, data-dependent mode. Full scans were performed at a resolution of 120,000 over the 380–1400 *m*/*z* range. The top 15 most intense multiple charged ions were isolated (1.2 *m*/*z* isolation window) and fragmented at a resolution of 30,000, with a dynamic exclusion set to 30.0 s.

### 2.4. Transcriptome Assembly, Quantification, and Annotation

Raw RNA-seq data for *P. punctata* were obtained from the publicly available Sequence Read Archive (SRA) under accession ERR14056194, originating from Biosample SAMEA114771597. Quality assessment was performed using FastQC [32], followed by the trimming of adapter sequences and low-quality bases using Trimmomatic (version 0.39) [33]. Cleaned reads were assembled into contigs using Trinity (version 2.2.1) [34] with default parameters. Potential coding sequences were predicted using TransDecoder (version 5.7.1) [35], also with default settings. Transcript abundance quantification was performed using Kallisto (version 0.46.1) with default settings to obtain Transcripts Per Million (TPM) values. The coding sequence predictions were annotated using DIAMOND BLAST (version 2.1.9) [36] against a custom protein database previously described in Barroso et al. (2025) [37]. This integrative database includes curated sequences from Cnidaria, antimicrobial peptide (AMP), and toxin-related databases, and manually annotated venom components. Transcriptome completeness was assessed using BUSCO (version 6.0.0) with the metazoa_odb10 lineage dataset, yielding 88.8% complete BUSCOs (67.0% single-copy, 21.8% duplicated), 6.7% fragmented, and 4.5% missing out of the 954 BUSCO groups searched.

### 2.5. LC-MS/MS Data Processing and Protein Identification

The RAW files generated were analyzed using the Sequest HT software package within Proteome Discoverer (v. 2.4.0.305, Thermo Fisher Scientific, San Jose, CA, USA). Protein identification was performed using Sequest HT against the obtained annotated transcriptome. The search parameters included a fragment ion mass tolerance of 0.1 Da and a parent ion mass tolerance of 10 ppm. Trypsin was specified as the digestion enzyme, allowing for up to two missed cleavages. Carbamidomethylation of cysteine was set as a fixed modification, while methionine oxidation was specified as a variable modification. Peptide and protein identifications were validated using Scaffold (version 5.3.3, Proteome Software Inc., Portland, OR, USA), applying a 0.1% false discovery rate (FDR) for peptides and a 1% FDR for proteins, with a minimum requirement of one uniquely identified peptide per protein. Protein probabilities were calculated using the Protein Prophet algorithm [38]. Peptide quantification was based on total spectral counts. Proteins supported by identification metrics that met these thresholds were considered to have significant peptide evidence and were retained for downstream analyses. Proteins with shared peptide evidence that could not be differentiated based solely on MS/MS data were grouped according to the principle of parsimony. Shared proteins between different tissues were visualized using Venn diagrams created with DeepVenn, available online: https://www.deepvenn.com (accessed on 24 January 2025).

### 2.6. Functional Annotation and Enrichment Analyses

Protein functional annotation was performed using InterProScan associated with Gene Ontology (GO) terms [39]. This analysis was carried out on both the complete set of validated proteins and a subset comprising uncharacterized proteins (e.g., hypothetical, predicted without annotation, or unnamed protein products).

In parallel, GO enrichment analysis was conducted. GO annotations were obtained through EggNOG-mapper, and enrichment testing was performed using the topGO R package (version 5.3.3) with the classic Fisher’s exact test. To enhance biological interpretability, only GO terms with more than three annotated proteins and at least two significant hits were retained for visualization. The most-enriched terms (top six per ontology: Biological Process, Molecular Function, and Cellular Component) were selected based on the highest −log_10_(*p*-value) scores. The GO.db package was used to retrieve ontology classifications. Results were visualized as horizontal bar plots using ggplot2 (version 3.5.2), with terms grouped and color-coded by ontology. Data preprocessing was conducted using the dplyr (version 1.1.4) and tidyr (version 1.3.1) packages.

Additionally, KEGG pathway enrichment analysis was performed to explore the pathway-level functional specializations among tissue-specific proteins. KEGG Orthology (KO) assignments obtained via EggNOG-mapper were used to associate proteins with pathways. Enrichment analysis was carried out using a custom R pipeline based on Fisher’s exact test, comparing the frequency of each KEGG pathway in each tissue-specific dataset (gonads, mantle, and oral arms) against the complete protein set. KEGG pathway descriptions were retrieved from the KEGG REST API. *p*-values were adjusted using the FDR method, and significantly enriched pathways (*p* < 0.05) were visualized using ggplot2. The enrichment plot displayed tissue-specific pathway enrichment based on −log_10_(*p*-value), with the dot size proportional to the number of tissue-associated proteins.

### 2.7. Identification of Putative Toxins and Phylogenetic Analysis of Jellyfish Toxins (JFTs)

Putative protein toxins were identified based on functional annotations, domain predictions, comparative sequence analyses, and bibliographic evidence. Due to the presence of incomplete or redundant transcript isoforms and inconsistencies between transcriptomic and proteomic datasets, protein sequences detected in the *P. punctata* proteome were mapped to their closest full-length homologs in public databases (UniProt and NCBI). This approach improved annotation confidence and enabled reliable downstream analyses. Notably, the transcriptomic and proteomic data originated from different individuals, further justifying the use of curated reference sequences for functional and phylogenetic inference.

Amino acid multiple sequence alignments (MSAs) were performed for jellyfish toxins (JFTs), incorporating *P. punctata* matched peptides, along with 12 additional JFT sequences from Scyphozoa and Cubozoa. Three-domain Cry toxins (3d-Cry) from *Bacillus thuringiensis* were used as outgroup sequences.

Alignments were generated using Geneious v11.1.5. and the MAFFT algorithm via the GUIDANCE2 webserver (https://guidance.tau.ac.il/, accessed on 31 January 2025), with 100 bootstrap replicates. Alignment columns with fewer than 17.65% informative residues were trimmed to improve phylogenetic reliability.

The refined MSA was submitted to ProtTest3 v3.4.2 [40] to determine the best-fitting amino acid substitution model based on the corrected Bayesian Information Criterion (BIC). Phylogenetic inference was conducted using the maximum-likelihood (ML) algorithm implemented in IQ-TREE v2.0.7 (http://www.iqtree.org/, accessed on 31 January 2025) under the WAG substitution model. Node support was assessed using 10,000 ultrafast bootstrap replicates, 10,000 replicates of the Shimodaira–Hasegawa approximate-likelihood ratio test (SH-aLRT), and an approximate Bayes test (aBayes).

### 2.8. Peptide Characterization and Antimicrobial Peptide (AMP) Prediction

The peptides identified in the dataset were evaluated for their potential to function as AMPs. Key physicochemical properties, including peptide length, net charge, isoelectric point (pI), molecular weight (MW), average hydropathicity (GRAVY), and Boman index, were calculated using an in-house Python script integrated with the ProtParam tool from the Expasy Proteomics Server [41].

To assess the AMP potential of each peptide, a combination of machine learning- and deep learning-based AMP prediction tools was employed, including AMPScanner vr.2 [26], PepNet [27], AI4AMP [25], and CAMPR4 with the random forest algorithm [24]. Although a precision-weighted average of AMP probability scores could improve prediction reliability by accounting for inter-model variability, reported precision values for these models vary considerably across studies [42,43,44]. Due to this inconsistency, we used a simple unweighted average of the probabilities from all models, acknowledging this as a limitation in the prediction approach.

Candidate peptides were filtered based on thresholds derived from the average physicochemical and predictive characteristics of AMPs reported in aquatic invertebrates, as described in our previous study [45]. Peptides were retained for further analysis if they met the following criteria:

- A positive net charge;
- A GRAVY value between −1.5 and +1.5;
- An average AMP prediction probability ≥ 0.7.

For structural characterization, the tertiary structures of the selected AMP candidates were modeled using two complementary tools: ColabFold [46], based on the AlphaFold2 algorithm [47], and PEP-FOLD4 [48], a tool specifically designed for the structural prediction of short linear peptides. AlphaFold2-based predictions were generated using five independent models per peptide, with 20 recycles to enhance structural accuracy. Predicted local distance difference test (pLDDT) scores and predicted template modeling (pTM) scores were retrieved to assess model confidence. PEP-FOLD4 was employed in parallel under default parameters for cross-validating structural predictions. All predicted structures from both methods were visualized and analyzed using ChimeraX [49] to identify secondary structural features. Structural patterns such as α-helices, β-sheets, and coil regions were annotated and compared with known AMPs to infer potential functional relevance. These structural models provide a basis for further studies on the mechanism of action and therapeutic potential of the predicted AMPs.

## 3. Results

### 3.1. Quantitative Correlation Between Transcriptomic and Proteomic Data

**Figure 2:**
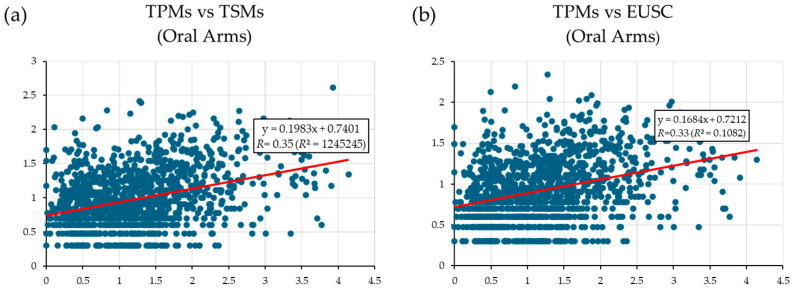
Scatter plots showing the correlation between transcript expression and protein abundance in oral arm samples. (**a**) Correlation between log10-transformed TPM values and TSMs, adjusted by adding +1. (**b**) Correlation between log10-transformed TPM values and EUSCs, also adjusted by +1.

The assembly produced 126,144 contigs (File S1), with 45,011 predicted coding sequences. There were 3160 transcript counts identified in the oral arms (out of 3184 total transcripts), 3064 in the gonads (out of 3086), and 2836 in the mantle (out of 2861). To assess the relationship between transcript expression and protein abundance, TPM values were correlated with two mass spectrometry-derived metrics from oral arm samples: Total Spectrum Matches (TSMs) and Exclusively Unique Spectrum Counts (EUSCs). Log_10_-transformed TPM, TSM, and EUSC values were used after adjusting by +1 to avoid undefined values. The results indicated moderate positive correlations, with Pearson’s correlation coefficients of *R* = 0.35 for TPM vs. TSMs and *R* = 0.33 for TPM vs. EUSCs (Figure 2).

### 3.2. Overview of Proteomic Data from P. punctata

**Figure 3:**
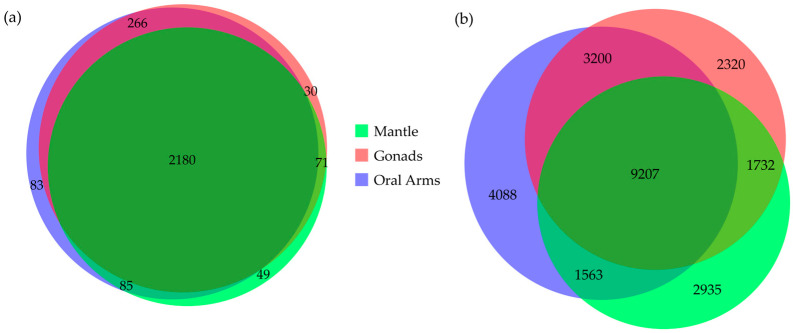
The area-proportional Venn diagram depicting the overlap of identified (**a**) proteins and (**b**) peptides across the three sample categories: mantle, gonads, and oral arms of *P. punctata*. The diagrams were generated using DeepVenn (https://www.deepvenn.com, accessed on 25 January 2025).

A total of 2764 proteins and 25,045 peptides were identified in the proteome of *P. punctata* (Table S1). Among these, 2180 proteins and 9207 peptides were present across all sample categories (Figure 3). Results show that oral arms contained the highest total number of proteins (2614) and peptides (18,058), followed by the gonads (2547 proteins and 16,459 peptides) and mantle (2385 proteins and 15,437 peptides). The oral arms also had the highest number of uniquely identified proteins (83) and peptides (4088), followed by the mantle (49 and 2935) and the gonads (30 and 2320).

The most abundant proteins identified were primarily housekeeping proteins, which are responsible for basic cellular functions (Table 1).

### 3.3. Functional Annotation and Enrichment Analysis of Tissue-Specific Proteins Identified in P. punctata

**Figure 4:**
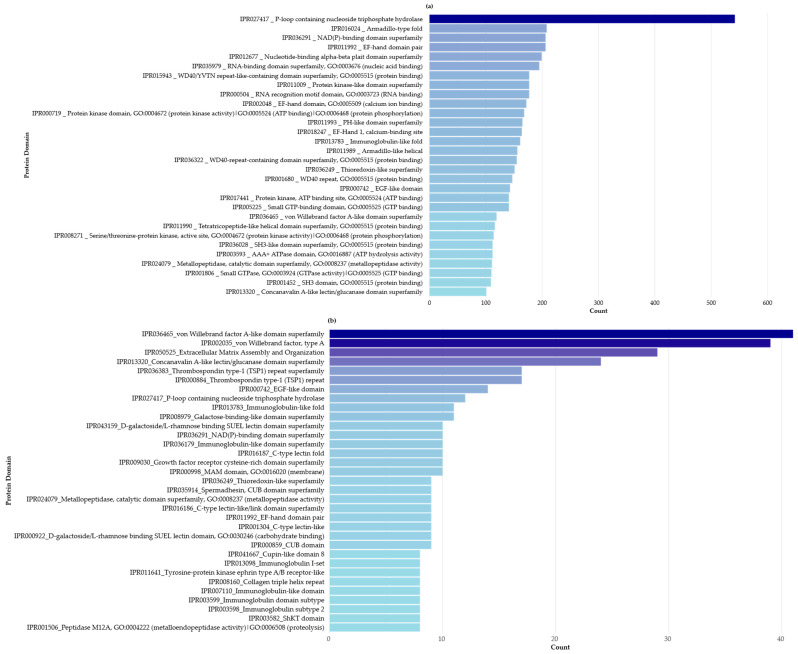
Most frequently identified InterPro domains in the *P. punctata* proteome with associated GO terms and descriptions: (**a**) top 30 domains across all identified proteins; (**b**) domains found in uncharacterized proteins only (minimum of 8 occurrences).

To elucidate the biological functions of tissue-specific proteins in *P. punctata*, functional annotation was performed using InterProScan associated with GO terms, revealing a diverse array of protein domains and functional sites (Figure 4). Notably, many previously uncharacterized proteins were found to contain conserved domains and associated GO terms, suggesting involvement in enzymatic activity, structural roles, and signal transduction. This analysis not only confirmed known cnidarian protein signatures but also uncovered potential novel functions among less-characterized sequences. The full InterProScan annotation dataset is provided in Table S2.

**Figure 5:**
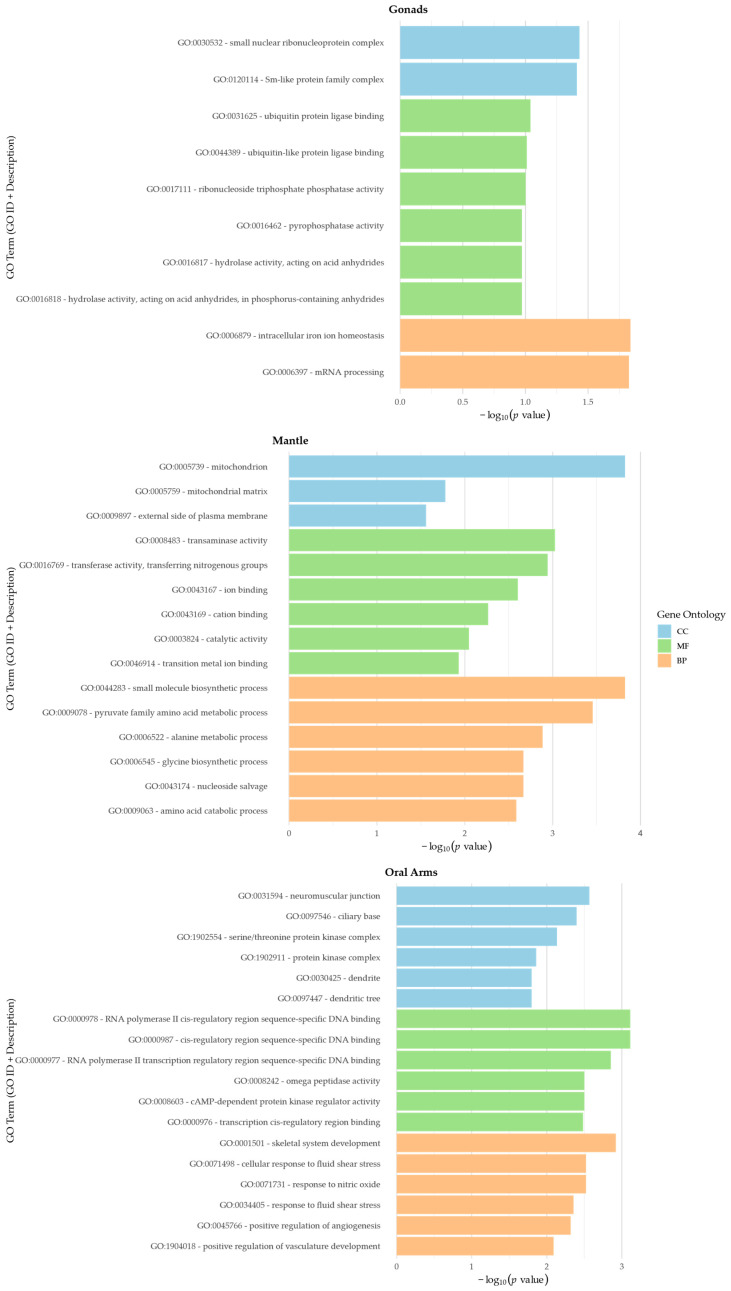
GO enrichment analysis of tissue-specific proteins in *P. punctata*. Bar plots show the top six enriched GO terms in each ontology, Biological Process (BP), Molecular Function (MF), and Cellular Component (CC), identified among proteins exclusive to the gonads, mantle, and oral arms. Enrichment significance is represented as −log_10_(*p*-value), and terms are color-coded by ontology. Only GO terms with at least three annotated proteins and two significant hits were included.

Subsequent GO enrichment analysis revealed the significant overrepresentation of specific functional categories within the tissue-specific protein sets of the oral arms, gonads, and mantle. The analysis covered the three GO domains, Biological Process (BP), Molecular Function (MF), and Cellular Component (CC), revealing clear functional differentiation across tissues (Figure 5). In the BP domain, gonads were enriched in terms related to mRNA processing and ion homeostasis; in the oral arms, for responses to mechanical and chemical stimuli, skeletal development, and angiogenesis; and in the mantle, for metabolic, catabolic, and oxidative stress-related processes. In MF, gonads were enriched in nucleotide hydrolysis and protein modification; the oral arms showed enrichment in transcriptional regulation, signaling, and proteolytic activity; and the mantle in catalytic functions, metal ion binding, and amino acid metabolism. For the CC domain, gonads were associated with RNA processing complexes and membrane-related structures; the oral arms with dendritic components and neuromuscular junctions; and the mantle with mitochondrial components and protein kinase complexes. The full GO enrichment dataset is provided in Table S3.

**Figure 6:**
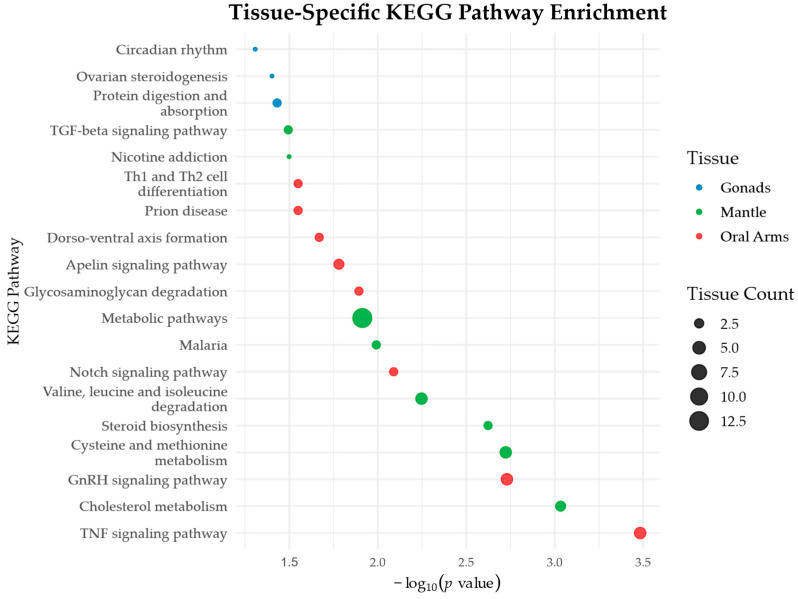
KEGG pathway enrichment analysis of tissue-specific proteins in *P. punctata*. Dot plot showing significantly enriched KEGG pathways (Fisher’s exact test, FDR-adjusted *p* < 0.05) among proteins exclusive to the gonads, mantle, and oral arms. The *x*-axis represents enrichment significance as −log_10_(*p*-value). Dot size indicates the number of proteins associated with each pathway, and colors distinguish the tissue of origin. Pathway descriptions were retrieved via the KEGG REST API.

To complement GO-based insights, KEGG pathway enrichment analysis was carried out using KO annotations to identify pathway-level functional specializations. Several pathways were significantly enriched in a tissue-specific manner, supporting the presence of metabolic and functional compartmentalization among the tissues (Figure 6). In the oral arms, enriched pathways included TNF signaling, GnRH signaling, Notch signaling, glycosaminoglycan degradation, and apelin signaling. Gonads showed enrichment in protein digestion and absorption, ovarian steroidogenesis, and circadian rhythm. In the mantle, enriched pathways included cholesterol metabolism; cysteine and methionine metabolism; steroid biosynthesis; valine, leucine, and isoleucine degradation; metabolic pathways; as well as malaria and TGF-beta signaling pathways. The complete KEGG enrichment results are provided in Table S4.

### 3.4. Identification and Comparative Analysis of Venom Components Across P. punctata Tissues

A total of 15 high-confidence toxin-related proteins were identified in the proteome of *P. punctata*, supported by consistent annotations across multiple databases (e.g., UniProt, Tox-Prot), the presence of conserved toxin-like domains, and homology to known venom proteins from other cnidarians (Table 2). An extended list, which also includes lower-confidence candidates with partial toxin signatures, is provided in Table S5.

**Figure 7:**
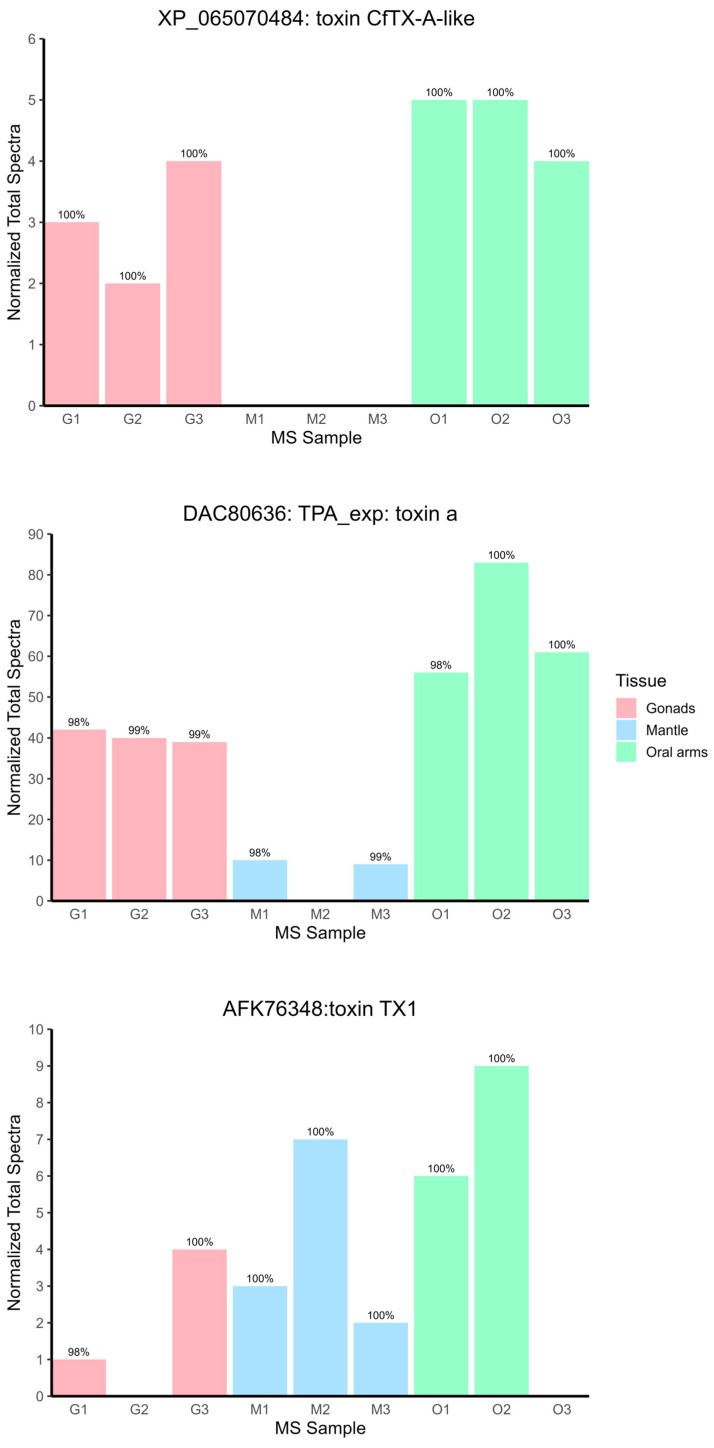
Normalized spectral abundance of JFT proteins identified in the *P. punctata* proteome. Bars represent normalized total spectra counts across different MS samples (oral arms, gonads, mantle). Only proteins with a minimum identification probability of 95% (as determined by the PeptideProphet algorithm in Scaffold) were included to ensure high-confidence identifications. TPA_exp: toxin a (DAC80636) was detected across multiple transcript entries; spectral values were aggregated and normalized accordingly. CfTX-A-like and TX1 toxins were each detected from a single unique transcript.

Among the high-confidence toxins, three were classified as JFTs: toxin TX1 from *A. aurita* (AFK76348); TPA_exp: toxin a from *Cassiopea xamachana* (DAC80636); and CfTX-A-like from *Rhopilema esculentum* (XP_065070484.1), which was not detected in the mantle tissue (Figure 7).

**Figure 8:**
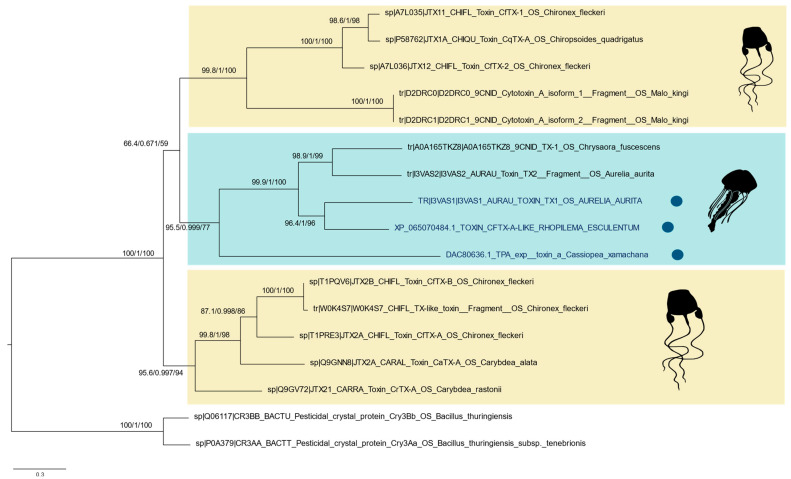
Maximum-likelihood (ML) phylogenetic tree of jellyfish toxins (JFTs). The tree was constructed using IQ-TREE based on amino acid sequences of best-matching reference proteins from UniProt and NCBI, with detected peptides from the *P. punctata* proteome mapped to these sequences. Node support values are shown for Shimodaira–Hasegawa approximate-likelihood ratio test (SH-aLRT), approximate Bayes test (aBayes), and ultrafast bootstraps. Colored dots indicate JFTs detected in the *P. punctata* proteome. Cubomedusae sequences are highlighted in yellow, and Scyphozoan sequences are highlighted in blue. Three-domain Cry (3d-Cry) toxins from *Bacillus thuringiensis* were included as outgroup sequences.

**Figure 9:**
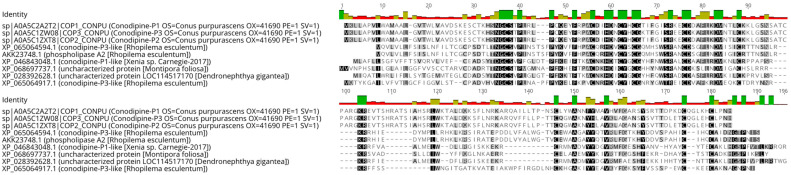
Multiple sequence alignment of a phospholipase A_2_ (PLA_2_) protein identified in the *P. punctata* proteome, aligned with conodipine-P3 sequences from *Conus purpurascens* (A0A5C2A2T2, A0A5C1ZW08 A0A5C1ZXT8), and top BLASTp hits from other cnidarian species. The *P. punctata* sequence corresponds to the best-matching reference protein from *Rhopilema esculentum* (XP_065064917.1). Conserved PLA_2_ motifs are highlighted.

These JFTs, which belong to Scyphozoa, are phylogenetically distinct from their Cubozoan counterparts (Figure 8). The corresponding multiple sequence alignment is available in Supplementary Material (File S2). Additionally, the identified phospholipase A_2_ conodipine-P3-like toxin (XP_065064594) exhibited conserved sequence motifs when aligned with the original conodipines from *Conus purpurascens* (Figure 9).

### 3.5. Peptide Characterization and AMP Prediction

A total of 25,045 peptides were identified in the *P. punctata* proteome, with sequence lengths ranging from 7 to 44 amino acids. Their physicochemical properties varied as follows: net charge (−15 to +3), isoelectric point (pI) (4 to 12), molecular weight (MW) (730 to 4610 Da), GRAVY (−3.3 to +2.8), Boman index (0.02 to 0.15), and average predicted AMP probability of 24%.

Based on selection thresholds derived from aquatic invertebrate AMPs [45], a total of 274 unique AMP candidates were retained, with 81 shared across all sample types, 29 exclusive to the gonads, 52 to the mantle, and 47 to the oral arms.

AMP potential was assessed using multiple machine learning and deep learning models (AMPScanner vr.2, PepNet, AI4AMP, and CAMPR4), and the final score for each peptide was computed as a precision-weighted average across all predictors.

The tertiary structures of all AMP candidates were predicted using both ColabFold with the AlphaFold2 model and PEP-FOLD4. While AlphaFold2 offers state-of-the-art protein structure prediction, it has recognized limitations for short, linear peptides, often resulting in a high number of disordered (random coil) predictions [47]. PEP-FOLD4, a tool specialized for de novo modeling of short peptides, was used to cross-validate these predictions. The predictions revealed notable differences between the two tools (Table 3). AlphaFold2 classified a high proportion (130/274) of peptides as random coils, whereas PEP-FOLD4 yielded fewer (45/274) in this category. Conversely, PEP-FOLD4 predicted a greater number of α-helical structures (203 vs. 140). Consistent predictions across both tools were found in 113 α-helical peptides, 1 β-sheet, and 28 random coils. These results suggest that AlphaFold2 may overestimate disorder in short peptides, reinforcing the importance of complementary prediction approaches.

To represent potential promising candidates, the top 10 AMPs were selected based on their physicochemical properties, the unweighted average of AMP probability across all models, and the structural consistency using both predictors (Table 4). The complete dataset, including AMP scores and structural predictions, is available in the Supplementary Material (Table S6 and File S3).

## 4. Discussion

### 4.1. Proteomic Analysis of P. punctata Tissues

The proteomic profile of *P. punctata* was constructed using the only available transcriptome of *P. punctata* as of January 2025. The dataset is labeled as “tentacle” but likely corresponds to oral arm tissue, since *P. punctata*, being a Rhizostomeae jellyfish, lacks marginal tentacles [50]. This discrepancy should be considered when analyzing the data, as the oral arms transcriptome is expected to provide a more accurate representation of the proteome for this specific tissue. Despite this, the approach still yielded significantly more and higher-quality results than using a general cnidarian-based database (results not shown), underscoring the need to improve the availability and quality of omics data for better species-specific analysis.

Oral arm samples indeed revealed a greater number of proteins and peptides identified. Interestingly, while the total protein coverage from the oral arms accounts for 95% of the total proteins identified, this value decreases to 72% when considering peptides instead. This difference could very likely be attributed to post-translational modifications (PTMs).

The most abundant proteins identified in the proteome of *P. punctata* were unsurprisingly mostly those with essential cellular functions: structural proteins such as myosin, filamin, actin, tubulin, and collagen, which contribute to maintaining cell shape, motility, and structural integrity [51,52]; proteins involved in energy production, like ATP synthase subunits alpha and beta, which play a fundamental role in cellular respiration and ATP synthesis for cellular metabolism [53]; proteins related to protein folding and stabilization, such as heat shock protein HSP 90-alpha-like, that ensure proper protein conformation and function, particularly under stress conditions, which is crucial for maintaining cellular homeostasis in response to environmental changes [54]; and proteins associated with vesicular trafficking, namely clathrin, which is vital for endocytosis and intracellular transport [55]. Additionally, there were proteins associated with cellular stress responses, like glutathione S-transferase 8 that participates in detoxification processes and protection against oxidative damage [56], and immune-related proteins such as macroglobulin, known for their role as broad-spectrum protease inhibitors [57]. Some of the most abundant identified proteins exhibit remarkable properties and are already widely applied across various fields. A notable example is collagen, which serves as a natural alternative to mammalian-derived collagen. Traditionally used in industries such as cosmetics and food production, mammalian collagen has faced growing concerns due to the risk of diseases like bovine spongiform encephalopathy and religious restrictions [58]. The highly conserved structure and sequence of fibrillar collagen make jellyfish-derived collagen a promising biomaterial, valued for its low immunogenicity and high biocompatibility [59].

### 4.2. Interpretation of Transcriptome–Proteome Correlation

The weak positive correlation between transcript and protein abundance suggests that while transcript abundance partially reflects protein detection, other biological and technical factors likely influence this relationship. This discrepancy is common, as proteomic datasets are often incomplete when compared to transcriptomic datasets [60]. The issue is possibly aggravated by the fact that transcriptomic and proteomic data were obtained from different specimens, introducing potential genetic and physiological variability. Additionally, environmental factors may contribute to these differences as well, as the transcriptomic data were derived from a wild specimen, whereas the proteomic data originated from aquarium-maintained individuals. This variation in habitat could lead to differences in gene and protein expression patterns [61,62]. Moreover, it is important to acknowledge the main factors of protein expression regulation, including post-transcriptional, translational, and protein-degradation processes [63]. Studies have shown that translation-related sequence features alone can account for up to 26% of the total variation of transcript–protein correlations [64]. Despite the moderate correlation observed, these findings provide valuable insights into the complexity of transcript–protein relationships, underscoring the importance of integrative approaches in transcriptomic and proteomic studies.

### 4.3. Tissue-Specific Functional Enrichment Reveals Metabolic and Regulatory Specialization

GO and KEGG enrichment analyses of *Phyllorhiza punctata* revealed distinct tissue-specific molecular profiles in the oral arms, gonads, and mantle, reflecting their specialized functions and highlighting the species’ ecological resilience and biotechnological potential.

In the gonads, enrichment was linked to RNA metabolism and nucleotide processing, supporting the high transcription and translation demands of gametogenesis in this fast-proliferating species [65]. Small nuclear ribonucleoprotein (snRNP) terms indicated active pre-mRNA splicing typical of dividing cells [66]. Pathways for ovarian steroidogenesis and circadian rhythm were also enriched, emphasizing hormone production and the temporal regulation of reproduction [67]. Metal regulation likely reveals the mechanisms of germ cells’ protection from oxidative stress [68].

The oral arms were enriched in signal transduction, transcriptional regulation, and mechanosensory response, consistent with their roles in prey capture and environmental sensing, which require neuromuscular coordination [69]. Immune and developmental pathways, including Th1/Th2 cell differentiation, TNF, apelin, and dorso-ventral axis formation, indicate immune defense and regenerative functions, aligning with their interaction with microbes and potential AMP production.

The mantle showed enrichment in mitochondrial components and metabolic pathways, supporting energy production and adaptation to environmental stress, traits likely contributing to the species’ invasive success. Metal ion binding and catalytic activity suggest roles in detoxification and microbial defense, as seen in other marine invertebrates [70].

### 4.4. P. punctata Venom Composition

Given that the diagnostic feature of cnidarians is the presence of cnidocytes, specialized cells containing cnidae, among which nematocysts are the venom-bearing type [3], it is important to acknowledge that all cnidarians have the potential for toxicity. This includes species not considered a threat to humans, such as the case of *P. punctata*. Therefore, analyzing the species’ venom proteome is crucial for gaining a comprehensive understanding of their full toxicological profile.

Among other toxins, three JFTs were identified in the proteome of *P. punctata*: toxin CfTX-A-like (XP_065070484) originally predicted from a genomic sequence of the rhizostomeaen jellyfish *R. esculentum*; toxin TX1 (AFK76348), previously identified from mRNA of *A. aurita* polyps; and TPA_exp: toxin a (GenBank ID: DAC80636.1), verified through proteomic analysis of the stinging-cell structures (cassiosomes) released in the mucus of *Cassiopea xamachana* [71]. These toxins are homologous to the JFTs, potent cytolytic toxins that disrupt cell membranes through pore creation. These were isolated initially from several species of box jellyfish and were linked with harmful stinging reactions to humans. For example, the toxins CfTX-1/2 and CfTX-A/B from the Australian box jellyfish *Chironex fleckeri* are highly cardiotoxic and hemolytic, respectively [72]. JFTs have also been identified in the tentacle and stinging-cell proteomes of Scyphozoa [71,73]; in the hydrozoan *Hydractinia symbiolongicarpus*, where their expression in nematocysts was confirmed at the proteomic level using immunohistochemistry [74]; and in Anthozoa, within the transcriptomes of Ceriantharia [75], though these findings still require validation through proteomic analysis. In the phylogenetic tree, the three JFTs are detected in the proteome of *P. punctata* clustered with other JFTs from Scyphozoa, forming a distinct clade separate from the two Cubozoan-specific clades. This phylogenetic pattern suggests that each group may have arisen through independent gene duplication and neofunctionalization events, potentially linked to lineage-specific differences in venom composition and toxic potency. A similar phylogenetic structure was observed in a broader study analyzing JFT distribution across 20 species representing all major medusozoan groups, but with more extensive taxon sampling [76]. Further studies will be required to understand the phylogenetic distribution of JFTs within scyphozoans and other cnidarians.

Additionally, a conodipine-P3-like derived from a genomic sequence of the scyphozoan *R. esculentum* was detected in the proteome of *P. punctata*. Conodipines are PLA2 toxins characterized from cone snail venoms [77]. However, similar toxins may be present in other venomous organisms, given that PLA2 enzymes have been convergently recruited into multiple insect orders, cephalopods, arachnids, reptiles, and cnidarians [78]. As revealed by the sequence alignment (Figure 7), several putative PLA2 toxins in Cnidaria with high homology to conodipines are known, containing the conserved catalytic domain residues, including the Asp/His dyad. Indeed, PLA2 activity has previously been detected in tissue homogenates of anthozoans, hydrozoans, scyphozoans, and cubozoans [79].

### 4.5. Potential AMPs Identified in P. punctata

Our previous study on the AMPs derived from aquatic invertebrates [45] provided valuable insights that enabled a more informed selection of candidate AMPs based on their most common physicochemical properties.

As a result, while the identification of both α-helical and β-like peptides among the candidates is an intriguing finding, it is not entirely unexpected. Generally, most AMPs adopt cationic amphipathic helices, a characteristic commonly associated with antimicrobial activity [80]. This trend is also observed in cnidarian AMPs, with peptides such as Aurelin from *A. aurita* [81], Arminin 1a from *Hydra* [82], Damicornin from *Pocillopora damicornis* [83], and AmAMP1 from *Acropora millepora* [84] all sharing cationic α-helices. However, some cnidarian AMPs adopt β-like structures instead, including defensin BDS-I from *Anemonia sulcata* [85] and Pd-AMP1 from the *Phyllogorgia dilatata* [86].

Additionally, no significant differences were found between tissues when comparing either the total number of peptides with the number of putative AMPs identified in each tissue or the number of exclusive peptides from each tissue with the number of exclusive putative AMPs identified in each tissue, which may suggest that *P. punctata* AMPs are not tissue-specific.

Furthermore, our integrative structure prediction approach revealed discrepancies between AlphaFold2 and PEP-FOLD4 outputs, particularly in the prediction of disordered regions. While AlphaFold2 tended to overpredict random coil structures, PEP-FOLD4 provided a more α-helix-rich profile. The overlap between methods in identifying consistent α-helical AMPs supports the utility of a dual-tool strategy for short-peptide modeling and strengthens confidence in the top candidate structures. Taken together, these findings support the presence of diverse and potentially bioactive AMP candidates in *P. punctata*, with structural traits aligned with known cnidarian antimicrobial peptides. This lays a strong foundation for future functional validation and potential biotechnological exploration.

## 5. Conclusions

Our findings highlight the largely untapped potential of cnidarians, particularly understudied species like *P. punctata*, as sources of toxins and AMPs. While *P. punctata* may not possess the potent toxin arsenal found in other jellyfish species, it still exhibits inherent toxicity. Moreover, as an invasive species, *P. punctata* demonstrates key features that favor its biotechnological exploration, including its adaptability to diverse environmental conditions, suggesting the production of bioactive compounds in response to environmental stressors, and its high reproductive capacity, which enables rapid biomass production for compound extraction. In this study, we identified several protein toxins in the species’ proteome, including JFTs and PLA2. Furthermore, using a proteome-wide and peptidome-integrated approach, we applied precision-informed AMP prediction combined with structural validation via AlphaFold2 and PEP-FOLD4. This pipeline enabled the identification of 274 candidate AMPs, including structurally diverse α-helical and β-sheet peptides. Importantly, no strong tissue-specific distribution of AMPs was observed, implying a systemic expression of these peptides in *P. punctata*. These findings lay a strong foundation for future studies, including in vitro assays to validate antimicrobial activity efficacy, assess cytotoxicity, and evaluate peptide stability and bioavailability. Together, these efforts will advance *P. punctata* as a suitable and versatile source of bioactive compounds for potential biotechnological applications.

## Figures and Tables

**Table 1:** Most abundant proteins identified in the proteome of *P. punctata*.

Accession	Protein Name	G	M	O	T
XP_065065861.1	myosin heavy chain, striated muscle-like	623	1724	706	3053
XP_065067169.1	filamin-A-like isoform X5	432	871	436	1739
ADR10434.1	non-muscle actin II	461	621	410	1492
XP_065065923.1	myosin-10-like isoform X1	367	272	400	1039
XP_065676232.1	tubulin beta chain	221	259	254	734
CAH3037106.1	unnamed protein product (actin)	190	323	170	683
XP_065054978.1	clathrin heavy chain 1-like	226	152	259	637
XP_065051959.1	probable glutathione S-transferase 8	235	193	202	630
XP_065060176.1	alpha-1-macroglobulin-like isoform X4	179	265	169	613
ACT_HYDVU	Actin, non-muscle 6.2	185	239	186	610
XP_065052735.1	uncharacterized protein LOC135681977	170	258	134	562
XP_065052612.1	ATP synthase subunit beta	146	282	131	559
XP_065065546.1	alpha-actinin-like	168	145	233	546
CAH3152489.1	unnamed protein product	193	80	248	521
XP_065065852.1	myosin heavy chain-like isoform X1	262	44	195	501
XP_065054022.1	collagen alpha-1(I) chain-like isoform X1	178	129	191	498
XP_065063720.1	heat shock protein HSP 90-alpha-like	184	150	154	488
XP_065051708.1	myosin light chain kinase, smooth muscle-like isoform X2	103	273	106	482
XP_065059929.1	ATP synthase subunit alpha, mitochondrial-like	120	250	108	478

G: gonads, M: mantle, O: oral arms, T: total.

**Table 2:** Major toxins identified in *P. punctata* and their corresponding total spectrum counts across sample categories (G: gonads, M: mantle; O: oral arms, T: sum of total spectrum counts). Underlined: JFTs.

Accession	Toxin Protein Name	G	M	O	T
DAC80636	TPA_exp: toxin a	121	23	200	344
XP_065065639	ras-related C3 botulinum toxin substrate 1	84	84	112	280
XP_065067572	venom factor-like	50	45	24	119
XP_065064594	conodipine-P3-like	34	25	45	104
XP_065067570	LOW-QUALITY PROTEIN: venom factor-like	37	18	19	74
XP_065054632	aflatoxin B1 aldehyde reductase member 2-like	22	30	10	62
CAB3985090	agrin-like	33	15	6	54
A0A7M5UUY9	BPTI/Kunitz inhibitor domain-containing protein	19	28	7	54
XP_065070484	toxin CfTX-A-like	18	2	28	48
XP_065055594	plancitoxin-1-like	10	11	13	34
QNH72454	toxin candidate	19	7	7	33
AFK76348	toxin TX1	5	12	15	32
XP_065063396	ADAM 17-like protease	10	9	13	32
XP_065065308	snake venom 5′-nucleotidase-like	4	7	10	21
XP_065071254	zinc metalloproteinase-disintegrin-like MTP8			3	3

G: gonads, M: mantle, O: oral arms, T: total.

**Table 3:** Summary table comparing AlphaFold2 and PEP-FOLD4 results.

Structure	No. Peptides (AlphaFold2)	No. Peptides (PEP-FOLD4)	Consistent Predictions (Both Tools)
α-helix	140	203	113
β-sheet	4	23	1
αβ-motifs	0	3	0
Random coil	130	45	28

**Table 4:** Top 10 AMP candidates filtered by physicochemical properties, AMP prediction scores, and structural consistency.

Protein Accession	Sequence	Predicted Structure (Alphafold2)	C Score	Avg Pred.	O	M	G
XP_065064294	QLGWCSTVKQAMKALCEK	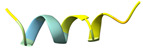	0.47	0.95		X	
XP_065069205	VCLIGAGNWGSAIAK	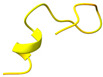	0.40	0.92	X	X	
XP_065058610	IGTKVLLKIYK	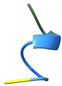	0.55	0.91			X
XP_065058870	IPTHAPYVIIGGGTASHAACR	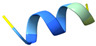	0.53	0.90		X	
XP_065062238	LPSSVIGSLIGK	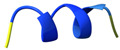	0.57	0.89			X
XP_065055185	GIRPAINVGLSVSR	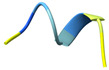	0.52	0.89		X	
XP_065059349	KPIGLCCIAPVLAAK	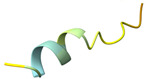	0.47	0.88	X	X	X
XP_065062330	LPVVTNQICSILNR	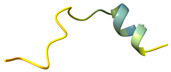	0.46	0.88		X	X
XP_065055150	GIQCLISVGLGTR	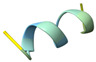	0.51	0.88			X
XP_065051697	DVMIIGPATVGGIKPGCFK	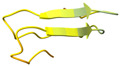	0.56	0.86			X

The table includes the protein accession number, peptide sequence, predicted structure, composite score (C Score), average AMP prediction probability (Avg pred.), and occurrence across the different sample types: oral arms (O), mantle (M), and gonads (G). C Score was calculated as (0.6 × N pLDDT + 0.4 × pTM) to balance local and global structure confidence.

## Data Availability

The mass spectrometry proteomics data have been deposited to the ProteomeXchange Consortium via the PRoteomics IDEntifications (PRIDE) Archive repository with the dataset identifier PXD066818. Additional data will be made available on request.

## Supplementary Materials

The following supporting information can be downloaded at: https://www.mdpi.com/article/10.3390/biom15081121/s1, Table S1: Comprehensive protein list identified from *Phyllorhiza punctata* tissues; Table S2: InterProScan annotation of identified proteins; Table S3: Gene Ontology (GO) enrichment analysis of identified proteins; Table S4: KEGG pathway enrichment analysis of identified proteins; Table S5: Putative toxins identified in the *P. punctata* proteome; Table S6: Predicted antimicrobial peptides (AMPs) in the *P. punctata* proteome; File S1: De novo transcriptome assembly of *P. punctata*; File S2: Multiple sequence alignment of jellyfish toxin family (JFT) proteins; File S3: Structural predictions of selected putative AMPs.
